# Correcting for Mortality Among Patients Lost to Follow Up on Antiretroviral Therapy in South Africa: A Cohort Analysis

**DOI:** 10.1371/journal.pone.0014684

**Published:** 2011-02-17

**Authors:** Gilles Van Cutsem, Nathan Ford, Katherine Hildebrand, Eric Goemaere, Shaheed Mathee, Musaed Abrahams, David Coetzee, Andrew Boulle

**Affiliations:** 1 Médecins Sans Frontières, Cape Town, South Africa; 2 Centre for Infectious Disease Epidemiology and Research, School of Public Health and Family Medicine, University of Cape Town, Cape Town, South Africa; 3 Department of Health, Provincial Government of the Western Cape, Cape Town, South Africa; McGill University Health Center, Canada

## Abstract

**Background:**

Loss to follow-up (LTF) challenges the reporting of antiretroviral treatment (ART) programmes, since it encompasses patients alive but lost to programme and deaths misclassified as LTF. We describe LTF before and after correction for mortality in a primary care ART programme with linkages to the national vital registration system.

**Methods and Findings:**

We included 6411 patients enrolled on ART between March 2001 and June 2007. Patients LTF with available civil identification numbers were matched with the national vital registration system to ascertain vital status. Corrected mortality and true LTF were determined by weighting these patients to represent all patients LTF. We used Kaplan-Meier estimates and Cox regression to describe LTF, mortality among those LTF, and true LTF. Of 627 patients LTF, 85 (28.8%) had died within 3 months after their last clinic visits. Respective estimates of LTF before and after correction for mortality were 6.9% (95% confidence interval [CI] 6.2–7.6) and 4.3% (95% CI 3.5–5.3) at one year on ART, and 23.9% (95% CI 21.0–27.2) and 19.7% (95% CI 16.1–23.7) at 5 years. After correction for mortality, the hazard of LTF was reversed from decreasing to increasing with time on ART. Younger age, higher baseline CD4 count, pregnancy and increasing calendar year were associated with higher true LTF. Mortality of patients LTF at 1, 12 and 24 months after their last visits was respectively 23.1%, 30.9% and 43.8%; 78.0% of deaths occurred during the first 3 months after last visit and 45.0% in patients on ART for 0 to 3 months.

**Conclusions:**

Mortality of patients LTF was high and occurred early after last clinic visit, especially in patients recently started on ART. Correction for these misclassified deaths revealed that the risk of true LTF increased over time. Research targeting groups at higher risk of LTF (youth, pregnant women and patients with higher CD4 counts) is needed.

## Introduction

Loss to follow-up (LTF) is recognised as one of the key challenges to evaluating the effectiveness of antiretroviral care in resource-limited settings. Reported rates of LTF vary considerably; one review of antiretroviral treatment programmes in Africa reported cumulative proportions of lost to care at two years ranging from 15% to 54% [1]. However, the vital status of those patients LTF is often unknown, and may include negative outcomes (such as mortality) and non-negative outcomes (such as transfers).

Several studies have traced patients lost to care to ascertain their true status. A recent systematic review of studies reporting outcomes on patients lost to care, who had been traced to ascertain their vital status, found that 20% to 60% had died and 37% could not be traced [2]. However, active tracing of all patients lost to care to ascertain vital status as part of routine monitoring and evaluation is generally not practical, and programmes commonly report outcome data simply as those remaining in care, thus aggregating death and loss to follow up as programme failures [3], [4].

However, beyond samples of patients who are traced for research purposes, the actual outcomes of a substantial proportion of patients remain unreported and unknown. High rates of LTF can result in programme reporting bias, due to inaccurate estimates of survival, and in biased estimates of risk factors for death and LTF, since patients lost to follow-up may be at high risk of death [5]. This is a concern both for individual clinical care and for programme evaluation, as unstructured interruption of treatment can lead to the development of drug resistance, [6] and there is uncertainty as to whether resources should be invested in defaulter tracing. Correctly detecting and minimising LTF is therefore a concern for health providers, programme planners and donors.

South Africa is the only country in sub-Saharan Africa with a well-functioning vital registration system, with more than 80% of deaths recorded in recent years [7], and this provides a unique opportunity to disentangle misclassified deaths and true loss to care. We report on mortality and LTF in patients in a primary care antiretroviral treatment programme in Khayelitsha, an area of Cape Town, before and after correction for vital status.

## Methods

### Study setting

The study included all treatment-naive adults initiated on ART at three public sector primary care clinics in Khayelitsha between March 2001 and June 2007 and followed up until January 2008. By the end of 2007, the service had cumulatively enrolled over 7000 adults onto ART. Antenatal HIV prevalence in 2007 was 32.7%. Patients with available civil identification numbers who were lost to follow-up were matched with the national death registry to ascertain their vital status.

Eligibility criteria for ART initiation in adults were CD4 cell count below 200 cells/mL and/or WHO stage IV with the exception of extrapulmonary tuberculosis. The standard first-line ART regimen was zidovudine (ZDV) and lamivudine (3TC) together with nevirapine (NVP) or efavirenz (EFV), with stavudine (d4T) replacing ZDV in late 2003, consistent with the South African national guidelines. A second-line regimen (ZDV, didanosine and lopinavir/ritonavir) is started after virologic failure is confirmed (two consecutive viral load results above 5000 copies/mL, in spite of enhanced adherence promotion after the first elevated result). The Khayelitsha ART programme has been described in detail elsewhere [8]–[10].

### Study design and key variables

In this prospective cohort study, patient outcomes were death, LTF or transfer to another facility. Patients were considered as LTF if their last clinic visit occurred at least 6 months before the end of the observation period (31 December 2007) and they were not known to have died or been transferred out. Definitions of loss to follow up vary considerably in the literature, from one day to more than one year [10]. Six months as a definition of LTF is increasingly used for analyses of African HIV treatment cohorts. This corresponds to at least two missed appointments for patients who are on a quarterly appointment schedule, which is usually the longest period between appointments in this setting. This duration has further been shown to perform best in terms of sensitivity and specificity from a multi-cohort analysis [11].

True LTF was defined for those patients with civil identification numbers as being lost to follow-up and not appearing in the national death registry during the first three months after LTF. Time in care was calculated from initiation on ART to the last recorded visit before 30 June 2007. Patients were followed in the analysis until mid-2007, and data until the end of 2007 were included to determine if patients met the LTF definition. Right censoring occurred at time of death or known transfer to another facility. Time to death in patients lost to follow-up was calculated from date of last visit until death if this occurred before database closure (25 January 2008), with right censoring of all patients who remained alive at this point. There was no uncensoring in either analysis.

### Data sources

The Khayelitsha programme serves as a sentinel monitoring site for the Western Cape Province. Data are captured routinely by health providers using structured clinical records, and these data are transferred to an on-site electronic patient information system by dedicated data clerks. Rule-based consistency and completeness checks are used to routinely audit the database.

South Africa's vital registration system has a high level of completeness and is rapidly updated. Citizens with identity documents have their details recorded on the national population register, and this register is updated immediately when a death is notified, before the detailed death notification form is processed by the central statistical office. Linkage to the population register therefore allows for a rapid assessment of mortality in those patients who have identity documents. There are strong administrative incentives for reporting deaths, and the completeness of death registration, especially in urban areas, has improved dramatically over the past decade. Sensitivity and specificity of the ascertainment of death through the national population registry has been assessed for this cohort at 90.5% and 99.9%, respectively [10]. This is consistent with estimates from local demographers as well as results from other studies using death registry linkages in South Africa [11].

### Statistical analysis

Descriptive analyses were based on percentages and frequencies for categorical variables, and medians and interquartile ranges (IQR) for continuous variables. To correct for unreported mortality among patients lost to follow-up, the updated vital status among lost patients for whom civil identification numbers were available was used to represent outcomes among all those lost to follow-up by generating a probability weighting (the ratio of all patients lost to follow-up to those lost for whom civil identification numbers were available). Lost patients with national death registry-updated vital status were assigned this weight, and all other lost patients were dropped from the analysis [3], [10]. We used weighted Kaplan-Meier estimates to describe time to loss to follow up and Cox regression models based on weighted and unweighted data to explore factors associated with loss to follow up and true LTF. Age, sex, and CD4 count were included in the multivariate models on a priori grounds. In addition, variables found to be associated with the outcome in the univariate analysis (p<0.25) were included in the multivariate model building process and retained if supported by likelihood ratio testing. Bootstrap confidence intervals for the weighted Kaplan–Meier failure estimates were calculated by sampling from the dataset with replacement 1000 times. Kaplan-Meier analysis and Cox proportional hazards regression were used to describe mortality and factors associated with death among the sub-sample of patients lost to follow-up for whom vital status data was available. A Wald test for linear hypotheses after estimation was used for ordinal variables (CD4 count, WHO stage, calendar year of initiation on ART, and drug regimen). All analyses were conducted using Stata statistical software, version 11.0 (Stata-Corp, College Station, Texas, USA).

### Ethics

The analysis of routine cohort data and the linkage to the national death registry were both approved by the University of Cape Town Research Ethics Committee. All data were anonymised prior to being made available for analysis.

## Results

### Cohort description

During the period under analysis, 6411 treatment-naive adult patients were initiated on ART, totalling 9846 person-years of observation. Annual enrolment on ART increased from 80 in 2001 to 2090 in 2006. The median age at enrolment on ART was 32 years (interquartile range [IQR] 28–39) and 68% were women (Table 1). Men were older than women (median age 36 vs. 31, p<0.001). At initiation of ART, patients truly lost to follow-up were younger (median age 31 vs. 33 years, p<0.001), had a higher CD4 count (median 116 vs. 99 cells/µL, p = 0.012), a lower WHO stage (34% vs. 37% with stage IV disease, p = 0.077), were more likely to be pregnant (6.2% vs. 2.0%, p<0.001), and initiated in later calendar years (p = 0.001) than patients not lost to follow-up; 37% of all patients were on treatment for tuberculosis and 8% had been exposed to prevention of mother-to-child transmission.

**Table 1:** Patient characteristics at initiation of ART.

		All	Not LTF*	True LTF**
**Total enrolled, n**		6411	5869	210
**Male sex, n (%)**		2067 (32)	1864 (32)	67 (32)
**Age, years**	**Women, median (IQR)**	31 (27–37)	31 (27–37)	30 (25–34)
	**Men, median (IQR)**	36 (31–42)	36 (31–42)	33 (30–38)
**Weight, kg**	**Tested, n (%)**	6198 (97)	5681 (97)	194 (92)
	**Median (IQR)**	59 (52–67)	59 (52–67)	59 (53–67)
**CD4 count, (cells/µL)**	**Tested, n (%)**	5963 (93)	5462 (93)	195 (93)
	**Median (IQR)**	99 (44–161)	99 (43–161)	116 (57–173)
**Viral load, (copies/ml)**	**Tested, n (%)**	3487 (54)	3154 (54)	126 (60)
	**Log10, Median (IQR)**	5.0 (4.6–5.6)	5.0 (4.6–5.6)	5.1 (4.6–5.5)
**WHO stage, n (%)**	**I**	359 (6)	327 (6)	20 (10)
	**II**	567 (9)	526 (9)	15 (7)
	**III**	3127 (49)	2855 (49)	104 (50)
	**IV**	2358 (37)	2161 (37)	71 (34)
**History of PMTCT, n (%)**		508 (8)	460 (8)	19 (9)
**Pregnant, n (%)**		139 (2)	120 (2)	13 (6)
**On TB treatment, n (%)**		2370 (37)	2159 (37)	75 (36)
**Antiretroviral regimen, n (%)**	**AZT/3TC/NVP**	470 (7)	429 (7)	22 (11)
	**AZT/3TC/EFV**	603 (9)	553 (9)	17 (8)
	**D4T/3TC/NVP**	2554 (40)	2357 (40)	81 (39)
	**D4T/3TC/EFV**	2748 (43)	2500 (43)	87 (42)
**Year of ART initiation, n (%)**	**2001**	82 (1)	76 (1)	1 (0)
	**2002**	205 (3)	195 (3)	4 (2)
	**2003**	389 (6)	354 (6)	11 (5)
	**2004**	1063 (17)	949 (16)	36 (17)
	**2005**	1643 (26)	1482 (25)	76 (36)
	**2006**	2090 (33)	1902 (32)	69 (33)
	**2007 (Jan–June)**	939 (15)	911 (16)	13 (6)

ART, antiretroviral treatment; LTF, Lost to follow-up; IQR, interquartile range; PMTCT, prevention of mother to child transmission; TB, tuberculosis; AZT, zidovudine; 3TC, lamivudine; NVP, nevirapine; D4T, stavudine; EFV, efavirenz.

* All patients not lost to follow-up, including deaths occurring within 3 months after loss to follow-up;

** Patients lost to follow-up with available civil identification number who were alive 3 months after being lost.

### Loss to follow up

**Figure 1:**
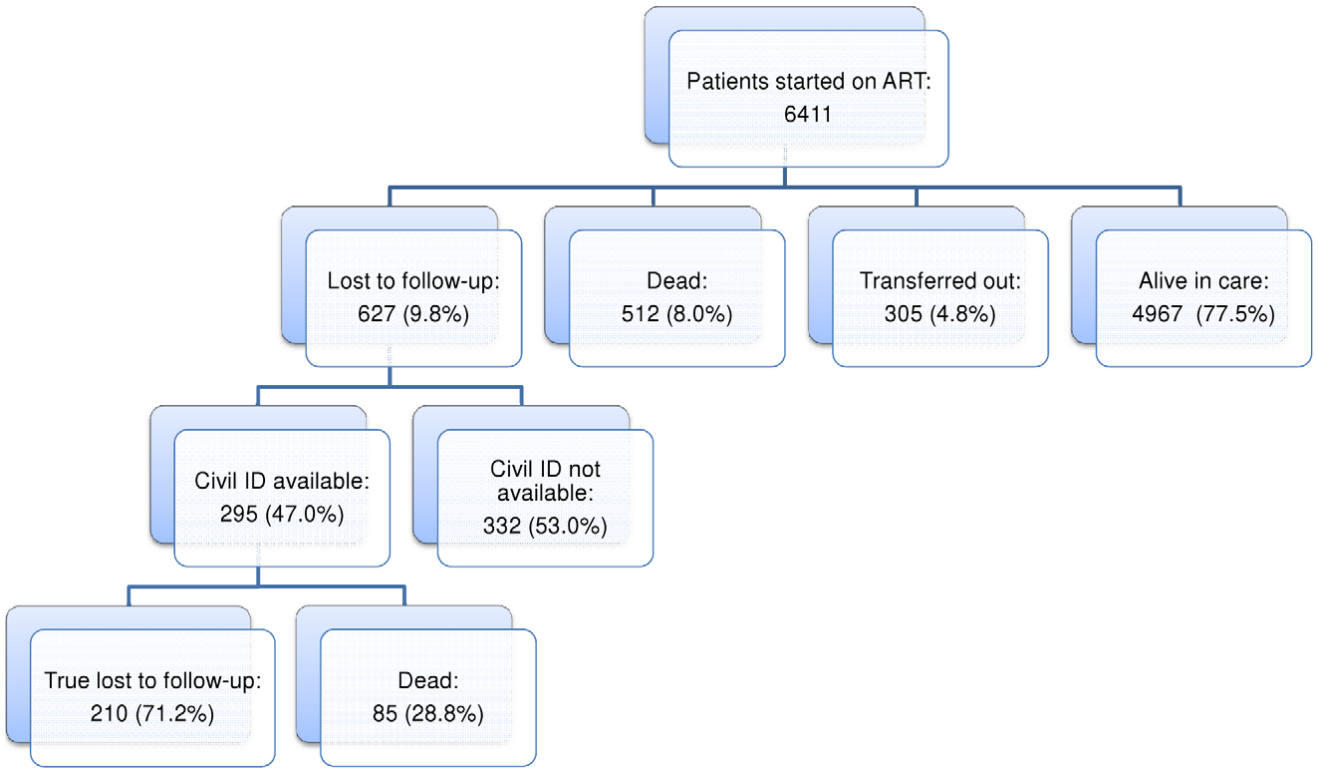
Summary diagram of patient outcomes.

At the end of June 2007, 4967 (77.5%) patients were alive and in care, 512 (8.0%) had died, 305 (4.8%) had been transferred to another facility, and 627 (9.8%) were lost to follow-up (Figure 1). Civil identification numbers were available for 295 (47.0%) of all patients lost to follow-up. Ascertainment of their vital status through the national death registry revealed that 85 (28.8%) had died within three months of LTF, 95 had died before 30 June 2007, and 109 before 25 January 2008. The only difference between patients LTF with and without available identification numbers was in the median CD4 count (respectively, 101 cells/µLµL, IQR 48–163 with, and 82, IQR 34–143 without identity documents, p = 0.007).

**Figure 2:**
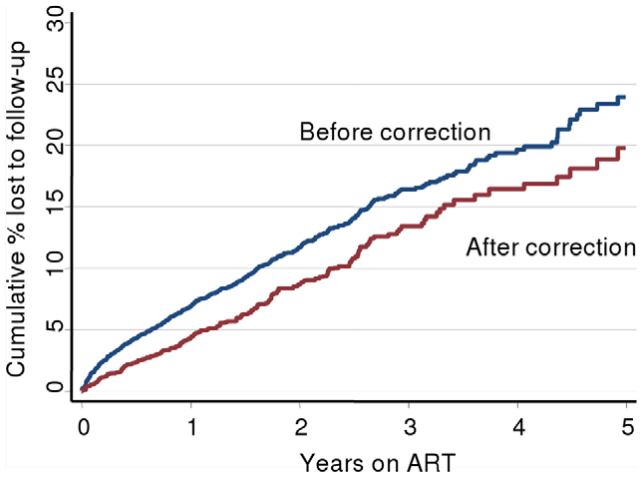
Cumulative probability of LTF before and after correction for mortality. Kaplan-Meier estimates of cumulative probability of loss to follow-up (LTF) before and after correction for mortality by ascertainment of vital status of patients lost to follow-up through the national vital registration system. Routine monitoring overestimated LTF by 4% at 5 years on ART (23.9 vs. 19.7%).

**Figure 3:**
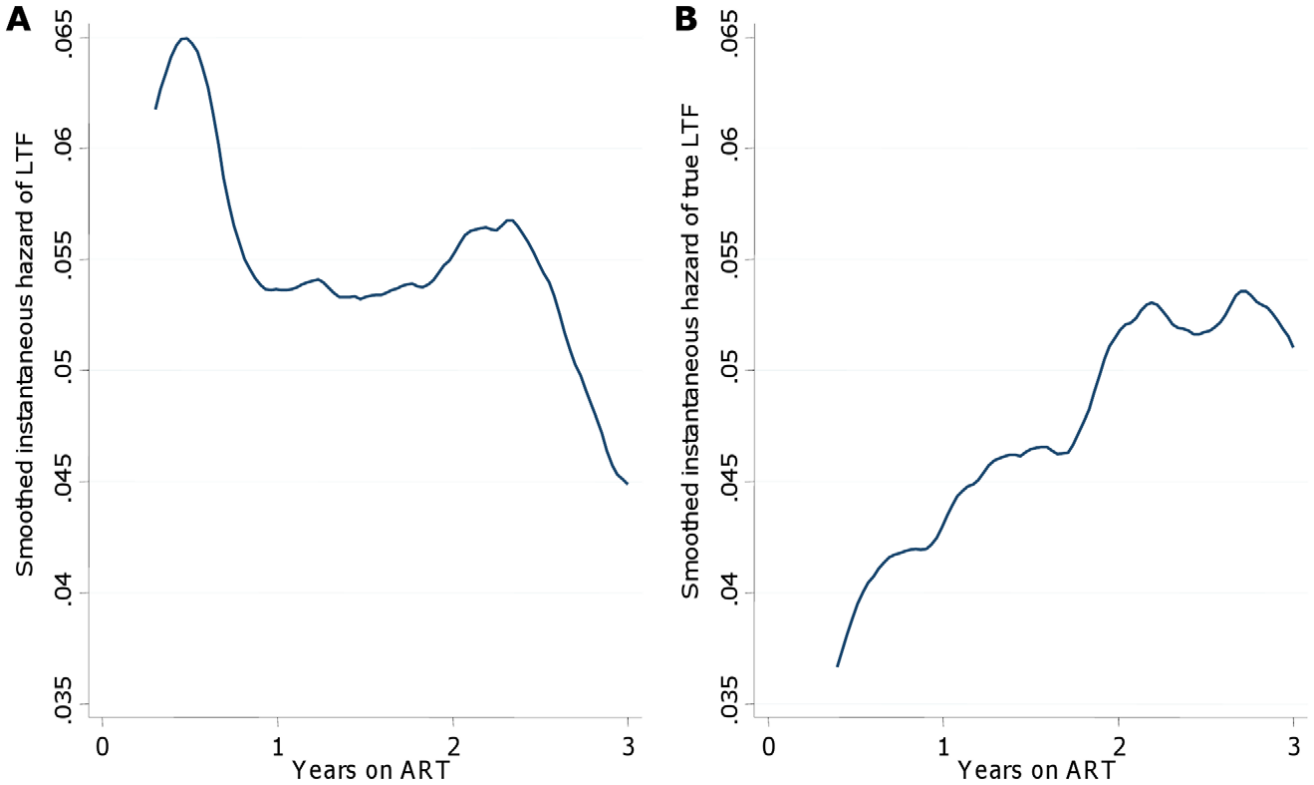
Smoothed hazard of loss to follow-up before and after ascertainment of vital status. Smoothed hazard estimates for loss to follow-up (LTF) before (A) and after (B) correction for mortality. Before ascertainment of vital status the hazard of LTF decreased over time on ART; after correcting LTF for mortality, the hazard of true LTF increased with time on ART.

**Figure 4:**
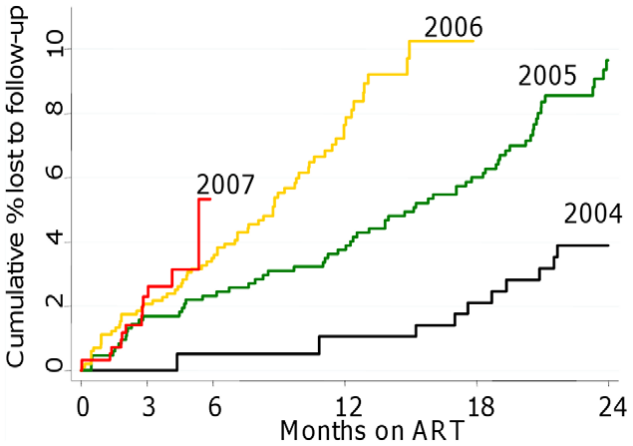
Cumulative probability of true LTF by year of initiation on ART. Weighted Kaplan-Meier estimates of true LTF by year of initiation on ART. True LTF increased and occurred earlier with each calendar year, as enrolment on ART increased.

Respective cumulative estimates of LTF before and after correction for mortality were 6.9% (95% CI 6.2–7.6) and 4.3% (95% CI 3.5–5.3) at one year on ART, 16.4% (95% CI 15.0–17.9) and 13.4% (95% CI 11.5–15.6) at three years, and 23.9% (95% CI 21.0–27.2) and 19.7% (95% CI 16.1–23.7) at 5 years (Figure 2). Before correction for mortality, the hazard of LTF decreased with increasing duration on ART (Figure 3). However, after correction, the hazard of true LTF was found to increase with time on ART. The cumulative proportion truly lost to follow-up at three months on ART increased from 0% in 2001–2004 to 1.7% in 2005, but then remained stable at 2.1% in 2006 and 2.3% in 2007 (Figure 4); true LTF at one year increased from 0% in 2001 to 2003, to 1.7% in 2004, 3.8% in 2005, and 7.6% in 2006.

Risk factors for LTF using routine data (prior to vital status linkage) were male sex (hazard ratio [HR] 1.32, p = 0.003), younger age (per 5 years older, HR 0.94, p = 0.027), lower weight (per 10 kg, HR 0.87, p<0.001) and increasing calendar year of initiation on ART (p<0.001; Table 2). After correction for mortality, younger age (HR 0.87, p = 0.017) and increasing calendar year (p<0.001) remained associated with true LTF, but there was no longer an association with sex or weight. Higher baseline CD4 count (p = 0.059) and pregnancy (HR 1.85, p = 0.072) were both associated with true LTF, whereas these factors were not associated with LTF when mortality was not taken into account. In univariate analyses, being on TB treatment at initiation of ART and antiretroviral regimens were both associated with LTF and true LTF. However, these associations were no longer present in multivariate analyses, suggesting confounding (by CD4 count for TB treatment and by calendar year for antiretroviral regimens).

**Table 2:** Cox proportional hazards models of factors associated with loss to follow-up before and after correction for mortality.

		Loss to follow-up (prior to linkage)						True loss to follow-up (after linkage)					
		Univariate			Multivariate			Univariate			Multivariate		
		HR	95% CI	p-value	HR	95% CI	p-value	HR	95% CI	p-value	HR	95% CI	p-value
Male gender		1.38	1.18–1.62	<0.001	1.32	1.10–1.58	0.003	0.94	0.70–1.26	0.673	1.10	0.80–1.53	0.553
Age (per five year increase)		0.98	0.93–1.02	0.320	0.94	0.89–0.99	0.027	0.87	0.79–0.97	0.010	0.87	0.78–0.98	0.017
Weight (per 10 kg increase)		0.86	0.80–0.92	<0.001	0.87	0.81–0.94	<0.001	0.98	0.87–1.10	0.712			
CD4 count (cells/µL)	0–49	0.94	0.75–1.18	0.793	1.00	0.74–1.35	0.223	0.49	0.33–0.73	0.002	0.62	0.41–0.93	0.059
	50–150	0.93	0.76–1.15		1.04	0.80–1.37		0.70	0.50–0.99		0.80	0.58–1.11	
	>150 (ref.)	1.00	-		1.00	-		1.00	-		1.00	-	
WHO stage	I (ref.)	1.00	-	0.821				1.00	-	0.034	1.00	-	0.240
	II	0.89	0.57–1.39					0.50	0.25–0.98		0.49	0.23–1.04	
	III	0.86	0.60–1.22					0.50	0.31–0.82		0.74	0.43–1.28	
	IV	0.84	0.59–1.21					0.48	0.28–0.80		0.85	0.48–1.51	
On TB treatment		1.37	1.17–1.61	<0.001				1.12	0.83–1.51	0.445			
Year of initiation of ART	2001/2/3 (ref.)	1.00	-	<0.001	1.00	-	<0.001	1.00	-	<0.001	1.00	-	<0.001
	2004	3.11	2.05–4.7		3.22	2.09–4.95		5.84	2.33–14.65		6.43	2.25–18.37	
	2005	6.00	3.92–9.19		5.71	3.66–8.92		10.58	3.85–29.06		10.17	3.24–31.89	
	2006	11.72	7.56–18.18		12.42	7.84–19.69		22.80	8.25–62.99		25.07	8.03–78.26	
	2007	12.90	7.60–21.88		14.75	8.35–26.07		23.17	7.28–73.77		28.09	7.92–99.59	
Viral load (log10 copies/µL)		1.09	0.96–1.25	0.207				1.09	0.86–1.37	0.486			
Exposure to PMTCT		0.81	0.61–1.08	0.153				0.86	0.54–1.38	0.535			
Pregnancy		1.60	1.01–2.53	0.043				3.02	1.76–5.2	<0.001	1.85	0.95–3.62	0.072
Antiretroviral regimen	AZT/3TC/NVP	0.89	0.63–1.25	<0.001				1.03	0.59–1.79	0.012			
	AZT/3TC/EFV	0.60	0.43–0.82					0.47	0.26–0.83				
	D4T/3TC/NVP (ref.)	1.00	-					1.00	-				
	D4T/3TC/EFV	1.60	1.34–1.91					1.21	0.88–1.67				

ART, antiretroviral treatment; WHO, World Health Organization; HR, hazard ratio; CI, confidence interval; PMTCT, prevention of mother to child transmission; TB, tuberculosis; AZT, zidovudine; 3TC, lamivudine; NVP, nevirapine; D4T, stavudine; EFV, efavirenz. P-values for CD4 count, WHO stage, year of initiation on ART and antiretroviral regimen are from Wald test for linear hypothesis after estimation. After correction for mortality, loss to follow-up was not associated with male gender or weight anymore, while new associations emerged with higher baseline CD4 counts and pregnancy at initiation of ART.

### Mortality of patients lost to follow-up

**Figure 5:**
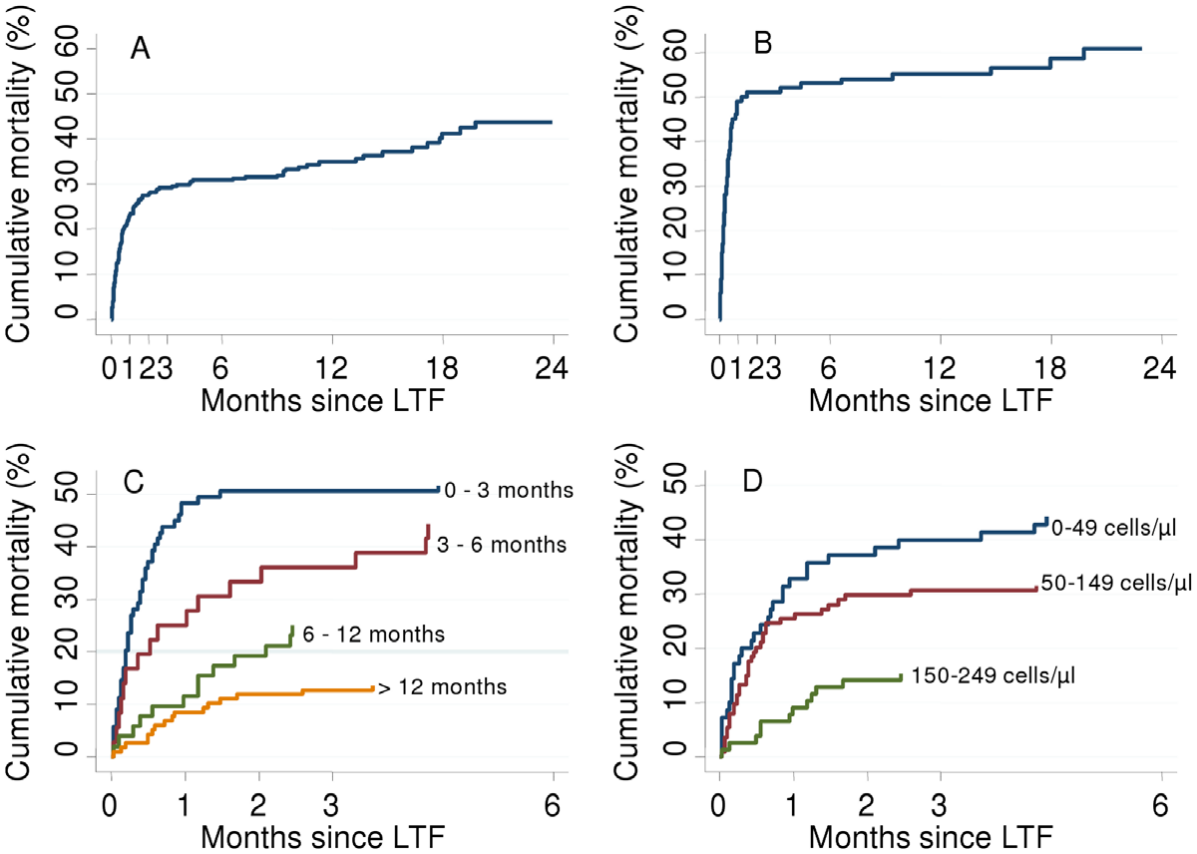
Cumulative mortality after LTF. These Kaplan-Meier graphs show cumulative mortality over time after the last recorded visit for patients originally classified as LTF in whom vital status could be ascertained through the national vital registration system: A - among all LTF; B - among LTF on ART for less than 3 months; C stratified by duration on ART at time of LTF; D stratified by CD4 cell count at initiation on ART. Patients recently started on ART and with lower CD4 counts had higher mortality after LTF. LTF, loss to follow-up.

Of the 295 patients LTF for whom vital status could be ascertained, 109 (36.9%) had died. Cumulative mortality of patients LTF at 1, 2, 3, 6, 12, 18 and 24 months after their last clinic visit was respectively 23.1%, 27.5%, 29.2%, 30.9%, 34.9%, 41.3%, and 43.8%. Thus, 78.0% (85/109) of deaths occurred within the first 3 months after their last clinic visit (Figure 5 A).

Mortality of patients lost to follow-up occurred predominantly in patients who had started ART recently, with 49/109 (45.0%) deaths in patients who were LTF when on ART for less than 3 months (Figure 5 B). For patients reported as lost during the first 3 months on ART, mortality at 3 months after their last clinic visit was stable at around 50% from 2005 to 2007.

Mortality of patients reported LTF at one month after their last clinic visit was, respectively, 48.3%, 25.0%, 11.5%, and 8.5% in patients on ART for 0–3 months, 3–6 months, 6–12 months and more than 12 months when lost (Figure 5 C). Patients lost to follow-up with a baseline CD4 count below 50 cells/µL were at increased risk of death (Figure 5 D). In multivariate analysis, baseline risk factors for death among patients lost to follow-up for whom vital status was available were lower weight (per additional 10kg, HR 0.75, p = 0.010), lower CD4 count (p = 0.011), and increasing calendar year of initiation (p = 0.012) (Table 3).

**Table 3:** Cox proportional hazards models of factors associated with mortality after reported loss to follow-up.

		Univariate			Multivariate		
		HR	95% CI	p	HR	95% CI	p-value
Male gender		1.69	1.16–2.47	0.007	1.14	0.74–1.76	0.559
Age (5 years older)		1.16	1.07–1.27	0.001	1.07	0.96–1.19	0.250
Weight (10 kg more)		0.68	0.57–0.82	<0.001	0.75	0.61–0.93	0.010
CD4 count (cells/µL)	0–49	3.40	1.89–6.12	<0.001	2.68	1.40–5.14	0.011
	50–150	2.19	1.23–3.88		1.81	0.97–3.38	
	>150 (ref.)	1.00	-		1.00	-	
WHO stage	I (ref.)	1.00	-	0.113	1.00	-	0.818
	II	2.94	0.76–11.37		1.75	0.33–9.27	
	III	3.18	0.99–10.18		1.83	0.42–8.0	
	IV	3.97	1.23–12.75		2.03	0.45–9.11	
On TB treatment		1.61	1.11–2.35	0.013			
Year of initiation of ART	2001–2003 (ref.)	1.00	-	<0.001	1.00	-	0.012
	2004	0.77	0.23–2.65		0.73	0.21–2.55	
	2005	1.67	0.59–4.69		1.92	0.67–5.51	
	2006	2.36	0.85–6.58		2.13	0.74–6.13	
	2007	4.59	1.54–13.71		4.22	1.25–14.21	
Viral load (log10 copies/µL)		1.18	0.81–1.74	0.391			
Exposure to PMTCT		0.30	0.09–0.94	0.038			
Pregnancy		0.15	0.02–1.08	0.060			
Antiretroviral regimen	AZT/3TC/NVP	0.23	0.05–0.94	0.002			
	AZT/3TC/EFV	0.71	0.28–1.82				
	D4T/3TC/NVP (ref.)	1.00	-				
	D4T/3TC/EFV	1.70	1.13–2.57				

ART, antiretroviral treatment; WHO, World Health Organization; HR, hazard ratio; CI, confidence interval; PMTCT, prevention of mother to child transmission; TB, tuberculosis; AZT, zidovudine; 3TC, lamivudine; NVP, nevirapine; D4T, stavudine; EFV, efavirenz. P-values for CD4 count, WHO stage, year of initiation on ART and antiretroviral regimen are from Wald test for linear hypothesis after estimation.

## Discussion

Our study presents data from one of the largest cohorts of patients lost to follow up in a resource-limited setting where linkages to national death registry allowed for ascertainment of vital status. We found that in the first few months on ART half of patients LTF had in fact died. True LTF, in which the majority of patients were alive when missing clinical appointments for six months or more, was nevertheless an important independent outcome, cumulatively accounting for a fifth of patients by five years on ART, and increasing in recent years among patients on ART for more than a year. Importantly, we found that subsequent to vital registry linkage, novel associations with true LTF emerged that were not apparent before correcting for mortality, including pregnancy and less severe immunodeficiency at ART initiation.

### High early mortality among those reported as LTF

High mortality in patients lost to follow-up has been described by other large ART programs in sub-Saharan Africa [12], often implying that patients lost to follow-up are at an increased risk of subsequent mortality. This study, however, demonstrates that in the early months on ART, mortality most likely preceded the missed appointments in patients who died and met LTF definitions. These patients more correctly represent misclassified deaths than patients lost to follow-up. Community-based retention strategies comprising early defaulter tracing might have very low efficiency if more than half the patients traced had already died. Conversely, increased clinical attention to and triaging of patients at high risk of death might decrease early mortality. Our finding that low baseline weight and CD4 count and recency of ART initiation are associated with higher mortality is consistent with other studies and points to the need to enrol patients earlier in their disease progression [10], [13]–[15].

### Increasing true LTF with duration on ART

The finding that, after correction for mortality, the rate for true LTF increases with duration on ART has implications both for patient care and cohort monitoring. This finding is consistent with previous studies that were not able to correct for misclassified deaths [16] and highlights the need for adapted counselling and adherence support interventions for patients on long-term ART. Possible reasons for this include treatment fatigue and improved immunological and clinical status leading to patients no longer seeing the need for medication.

### Increasing true LTF with calendar time

The overall proportion of people lost to follow-up in our cohort is low compared to that reported in many other studies [1]–[2], [16], but increased as the programme expanded. The most likely explanation is that major scale up of enrolment onto ART has come at a cost, with reduced quality of counselling, less intensive clinical care, and less support provided to individual patients. Initially, access to ART care was limited and patients were required to meet stringent criteria before being eligible for treatment [17]; these patients who successfully managed to access treatment in the early years may differ from those accessing care in more recent years. Decentralisation of care to peripheral sites [18] and community support have been found to be associated with reduced LTF and mortality [19], and such models could be explored as cohorts expand.

### Comparing mortality in those lost to follow-up with other programmes

Overall, the majority of deaths occurred among patients who were being followed in care (8%) compared with those who were truly lost to follow-up (3%). This is in contrast to other programmes in Africa, where most mortality has been reported to occur among patients who were reported as lost to follow-up [2]. In the absence of verification of vital status through death registration, our cohort would have underestimated cumulative mortality at one year by around 3% [10], with one third of deaths being misclassified as patients lost to follow-up. This contrasts sharply with a recent study from Botswana that estimated that 60% of deaths in the first year were among those lost to follow up [5]. The most likely explanation for this difference is a substantially higher proportion of early reported LTF and corrected mortality (after tracing) in this cohort (17% and 17% at one year in the Botswana cohort vs. 7% and 10% in our cohort). Other notable differences include a smaller sample size (410 vs. 6411) and follow-up duration (median follow up time 44 weeks vs. 5 years) and a sicker population accessing ART (median CD4 81 vs. 99 cells/mm^3^). Our data are, however, comparable to other data in South Africa [19].

### Risk factors for loss to follow up

Cohort monitoring helps in the identification of potential risk factors for true LTF based on patient characteristics. This allows programme implementers to focus counselling on various high-risk groups such as, in this instance, pregnant women and adolescents. A more thorough understanding of reasons for defaulting requires tracing of patients. Such studies have identified a number of risk factors including cost of treatment [20], improved or deteriorated health [2], stigma [21], fear of drug side-effects [22], loss of hope in medications [23], substance misuse [23], lack of money for transport [10], [23] and work or family responsibilities [23]. A recent modelling study reported that implementing a number of approaches to overcome commonly reported barriers to remaining in care such as removal of user fees and support with transport costs would be cost-effective [24]. However, there remains a paucity of implementation studies to limit defaulting in resource-limited settings. This is in contrast to adherence interventions, for which a multitude of randomised trials have been done or are underway and focus on adherence in patients who remain in care, often predominantly soon after initiating ART [25].

At the end of 2009 the World Health Organization recommended that ART initiation be started earlier in resource-limited settings [26]. In line with these new recommendations South Africa recently changed the eligibility criteria for ART to include all pregnant women and patients diagnosed with tuberculosis who are HIV infected and have a CD4 count below 350 cells/µL [27] compared to only patients with a CD4 count below 200 cells/µL. Our findings that both pregnant women and patients with higher CD4 counts are at a greater risk of leaving care point to the need for adapted adherence support for these groups. A previous study from the same setting has raised similarly concerns about the retention in care of pregnant women who are started on ART [28].

### Strengths and limitations

Our study has several strengths and limitations. Representative sampling of patients lost to care to verify vital status has been proposed as a way to obtain accurate mortality estimates [4]. We used death registry linkages to estimate true LTF estimates in South Africa. Our finding that mortality among those lost to care increased over time cautions against the use of cross-sectional tracing studies at a one point in time for correcting cohort mortality. Tracing studies are, however, able to collect first-hand accounts of reasons for loss to care besides mortality, which can be used to inform programme design and appropriate interventions. Both approaches are subject to potential biases: in the case of sampling, there may be differences between those who are traced and those who could not be traced; in the case of vital registration linkages, there may be differences between those with and without civil identification numbers. The slightly lower median CD4 count in patients without civil identification numbers suggests that mortality among LTF might be underestimated in this analysis.

Recent studies have highlighted the fact that a proportion of patients who are LTF in fact interrupt treatment for transient periods, returning at a later date [29]. We are unable to report on rates of interruption in our cohort. Our approach however avoids the possibility of double counting patients who were LTF more than once by excluding from our definition of LTF patients initially lost to follow up who later returned to care.

Another limitation, from a programme perspective, is that this study has focused only on LTF among patients who had initiated ART. High mortality and LTF has also been documented among patients waiting to be placed on ART [30], and similar studies are needed to explore risk factors for such pre-ART defaulting from care to help develop strategies to retain people in care prior to ART initiation [31].

Finally, it should be noted that this study was done in a peri-urban township setting with a high patient load, a high incidence of tuberculosis, and a high prevalence of HIV. The described cohort is representative of many scale-up treatment settings in South Africa and beyond. The increase with time in LTF is shared by a number of cohorts in South Africa [14], and the key findings from this analysis are of potential relevance to these and other cohorts in the region. Service and patient factors relating to loss to follow-up and the completeness of mortality registration are however affected by context, highlighting the importance of exploring the extent of and associations with true loss to follow-up in different settings.

### Relevance for policy and practice

As ART programmes worldwide grow larger, often with limited resources, increasing numbers of patients are lost to follow-up. Knowing the vital status of these patients is essential to design interventions to prevent loss to follow-up or to return these patients to care. First, this study shows that a large proportion of patients LTF during the first three months after starting ART died shortly after their last visit. There is thus a need to improve detection and care of patients at high risk of death during the first months on ART, e.g. by increasing clinical monitoring and the frequency of clinical visits for patients with low CD4 counts. In addition, these findings support earlier initiation of ART to prevent early mortality. Secondly, the finding that true LTF increased with time on ART confirms the need to develop adherence support strategies adapted to long-term ART. Currently, most ART programmes in resource-limited settings focus almost exclusively on early adherence support. And thirdly, this study confirms that younger age, pregnancy, and being started on ART with a higher CD4 count are risk factors for LTF. Programmes must be adapted to provide targeted support to youth and pregnant women, while more research is needed into adherence support that addresses the needs of patients with higher CD4 counts.

### Conclusions

Our study has demonstrated how the failure to verify deaths in the early months on ART impacts on the reported LTF on ART, and how, after correction for this misclassification, true loss to follow-up is increasing with both calendar time and duration on ART. The risk factors for true loss to follow-up identified by our study—higher CD4 and pregnancy— are likely to become more prevalent as countries adopted the latest WHO guidelines. This highlights the need for efficient real-time cohort monitoring and patient tracing as well as the development and implementation of interventions to reduce loss to follow-up among those at greatest risk. Finally, more must be done to prevent early mortality. The majority of ART programmes are unable to undertake such extensive monitoring as presented here; such deaths are wrongly misclassified as LTF, and therefore essentially invisible. Intensified clinical monitoring is needed for patients at high risk of death during the first months on ART.
